# Adaptation of the INTERGROWTH-21st neurodevelopment assessment (INTER-NDA) to the context of the English-speaking Caribbean

**DOI:** 10.1186/s12887-021-03039-7

**Published:** 2022-01-04

**Authors:** Randall Waechter, Roberta Evans, Sean Hanna, Toni Murray, Cassandra Mobley, Stephanie Holmes, Rashida Isaac, Rebeca Wolfe, Elbernezer Andrew, Barbara Landon, Michelle Fernandes

**Affiliations:** 1 School of Medicine, St. George’s University, True Blue, Grenada West Indies; 2 The Windward Islands Research and Education Foundation, True Blue, Grenada West Indies; 3 Psychological Services Center, St. George’s University, True Blue, Grenada West Indies; 4 National Institute of Health Research Biomedical Research Centre & Faculty of Medicine, Department of Paediatrics, University of Southampton, Southampton, UK; 5 Nuffield Department of Women’s & Reproductive Health, John Radcliffe Hospital, University of Oxford, Oxford, UK

**Keywords:** INTER-NDA, Neurodevelopmental assessment, Early childhood development, Child assessment, Assessment translation

## Abstract

**Background:**

Adaptation of standardized early child development (ECD) assessments to low- and middle-income countries can be challenging because of culture-specific factors relating to language, content, context, and tool administration, and because the reliance of these tests on specialist healthcare professionals limits their scalability in low resource settings.

**Methods:**

We report the cross-cultural adaptation of an international, standardized ECD instrument, the INTERGROWTH-21st Project Neurodevelopment Assessment (INTER-NDA), measuring cognitive, language, motor and behavioural outcomes in 2-year-olds, from a UK-based English-speaking population to the English-speaking Caribbean. Children aged 22-30 months were recruited from a pre-existing randomized controlled neurodevelopment intervention study in Grenada, West Indies.

**Results:**

Eight of 37 INTER-NDA items (22%) were culturally and linguistically adapted for implementation in the Caribbean context. Protocol adherence across seven newly-trained non-specialist child development assessors was 89.9%; six of the seven assessors scored ≥80%. Agreement between the expert assessor and the non-specialist child development assessors was substantial (κ = 0.89 to 1.00 (95% CI [0.58, 1.00]). The inter-rater and test-retest reliability for non-specialist child development assessors was between κ = 0.99 -1.00 (95% CI [0.98, 0.99]) and κ = 0.76 - 1.00 (95% CI [0.33, 1.00]) across all INTER-NDA domains.

**Conclusions:**

The current study provides evidence to support the use of the adapted INTER-NDA by trained, non-specialist assessors to measure ECD prevalence in the English-speaking Caribbean. It also provides a methodological template for the adaptation of child developmental measures to cultural and linguistic contexts that conform to the cultural standards of the countries in which they are utilized to aid in the measurement of neurodevelopmental impairments (NDIs) in a variety of global clinical settings.

**Supplementary Information:**

The online version contains supplementary material available at 10.1186/s12887-021-03039-7.

## Background

An estimated 249.4 million children under the age of five years, in low and middle-income countries (LMICs), are at risk of failing to achieve their full neurodevelopmental potential [18, 23]. There is considerable evidence of higher incidence and prevalence of neurodevelopmental impairment in LMICs compared to high-income countries [9, 18, 23]. The measurement of children at risk of neurodevelopmental impairment (NDI) is essential to providing interventions to children at highest risk, especially in LMIC settings where healthcare resources are often limited [14]. Moreover, quantification of NDI burden in and across populations is the first important step in measuring its long-term impact and evaluating the effectiveness of intervention strategies. Nevertheless, epidemiological data on normative child development are sparse or non-existent in many parts of the world, particularly in those areas where children are most at risk [3]. This creates challenges for early child development (ECD) surveillance in LMICs, where ECD outcomes in children from disparate geographical and/or cultural contexts are evaluated using instruments that have not been subjected to a rigorous standardization, adaptation and cultural-customization process for use in these settings. This is particularly significant in ECD assessments because social, cultural and language-related factors can adversely affect a child’s understanding of a test item and his/her subsequent performance on the ECD measure [30]. The use of inadequately adapted ECD assessment tools, at a population-health level, could result in over- or under-estimation of the prevalence of NDIs and, at an individual level, result in the misclassification of children as being at risk (or not) of NDIs. The life-course and public health consequences of either outcome are significant.

It is important to have a reliable ECD assessment tool that can be utilized to screen and assess the neurodevelopment of children residing in LMICs, as it enables healthcare systems residing in LMICs to: (1) conduct primary screenings, such as those held at daycare centers or local schools, to identify children at risk for NDIs; (2) refer children identified to be at risk for NDIs to primary and secondary healthcare settings, such as their primary healthcare provider or local rehabilitation clinic, respectively; and (3) assess the changes in functioning associated with newly-developed interventions that have been implemented either as a part of community-based or institution-based programs that aim to reduce the prevalence of NDIs within the country.

The importance of cross-cultural adaptation of ECD instruments, their adequate translation in the context of colloquialisms and accents; and the robust evaluation of adapted instruments has been highlighted in the World Bank’s Toolkit for the assessment of ECD in children aged under five years in LMICs [11, 12]. The Toolkit encourages cross-cultural adaptations because the data from the ECD assessments can provide tremendous developmental insight in areas where formal testing is unavailable, or rarely practiced. Measures that are not amended tend to demonstrate biases and poorer performance levels because some items do not function in the same manner across cultures [11]. Moreover, the interpretation of unfamiliar phraseology and its linkage to familiar lexical items to express roughly the same concept, are executive functions that can only be expected to emerge during mid to late childhood [10]. It is widely acknowledged that sociolinguistics is critically important when administering a standardized assessment within a culture, including within the same language, as pronunciation, tonality, sentence placement and the words themselves are very important in language processing [15].

One region in which there is limited data on ECD is the 700-island Caribbean basin with a regional population of 44 million [34]. Of the 33 countries and individual territories in the region, ten of these are classified as LMICs [38]. Previously, tropical infections represented the greatest disease burden in the region, however in recent years the burden has shifted to chronic diseases (during adulthood) and ECD disorders, including NDIs (during childhood) [27]. Importantly, the few coordinated efforts to assess individual or groups of young children in the Latin American and Caribbean region suggest that approximately 15% of children are at high risk of NDIs [23]. Despite this, there are, to our knowledge, no ECD tools adapted to and standardized in the cultural and linguistic context of the English-speaking Caribbean. We hypothesize that there are no culturally-adapted ECD assessments specifically for children living in the Caribbean because the region’s residents speak dialects of English, French or Spanish. European and American instruments have been directly exported there without a consideration of how linguistic and cultural differences between these disparate geographical and social populations might influence the test delivery and children’s performance.

The current study addresses this methodological gap in ECD measurement in the Caribbean by, for the first time, (1) adapting (from British to Caribbean English) and culturally customizing a rapid, multi-dimensional, international, standardized ECD instrument, *The INTERGROWTH-21st Neurodevelopment Assessment (INTER-NDA)*, for the measurement of cognitive, motor, language and behavioral outcomes in young Caribbean children, (2) comparing the psychometric properties of the adapted to the original tool and (3) evaluating the ability of non-specialist child development assessors to administer and score the adapted version of the INTER-NDA.

## Methods

### Study location

The study was carried out in the tri-island nation of Grenada, West Indies. Grenada has been an independent English-speaking nation since 1974. Twenty-three percent of Grenada’s population is under 15 years of age [17]. Throughout the region, approximately half of all households are headed by a single parent [1]. Despite its ranking as an upper middle-income country, approximately a third of its 112,000 residents live in poverty. Unemployment is reported at 20% and the nation’s debt ratio of 110% GDP ranks second worldwide [37]. The gross national income per capita was reported at $11,650 United States Dollars in 2014 [37]. Ethnically, more than 80% of the population identifies as Afro-Caribbean; the remainders are of mixed, or East Indian ancestry [5].

### Child development assessors sample

The characteristics of the seven non-specialist child development assessors are described in Table 1. All of the assessors were female and native Caribbean-English speakers. Five of the assessors were Caribbean nationals and two were American citizens. None of these assessors were psychologists or healthcare professionals, and none had a formal education beyond an undergraduate degree. Their income, representative of their socioeconomic status, was equivalent to Grenada’s 2014 gross national income per capita at $11,750 United States Dollars [37], adjusted for inflation.

**Table 1:** Characteristics of non-specialist child developmental assessors (*n* = 7)

Characteristic	N (%) or Mean (SD)
Age	29.8 (10.8)
Sex (Female)	Female – 7 (100%) Male – 0 (0%)
Number of years of Education	22.3 (3.1)
Native language (Caribbean-English)	7 (100%)
Ethnicity	Caribbean - 5 (71.4%) American – 2 (28.6%)

### Inter-rater and test-retest reliability sample – child participants

To evaluate inter-rater and test-retest reliability of the adapted INTER-NDA, each of the seven non-specialist child developmental assessors assessed three children, for a total of 21 child participants. Children ranged from 22 to 30 months old and were recruited from the “Saving Brains Grenada” project – a randomized controlled trial study assessing the impact of a Conscious Discipline intervention on reducing corporal punishment rates and improving neurodevelopmental outcomes among Grenadian children [22]. The 21 children included in the assessor training were recruited from four day-care centers around the capital city of St. George’s and were randomly assigned to a child development assessor for evaluation. The majority were female (52.4%), and all the children were Afro-Caribbean and spoke English as their native language (Table 2).

**Table 2:** Characteristics of inter-rater and test-retest reliability sample of child participants (n = 21)

Characteristic	N (%) or Mean (SD)
Child Sex	Female – 11 (52.4%) Male – 10 (47.6%)
Child Ethnicity (Caribbean)	Afro-Caribbean – 21 (100%)
Child Native language Child Age (Range, months)	English – 21 (100%) 22 – 30

### Internal consistency sample – child participants

To evaluate the Internal consistency of the adapted INTER-NDA, *n* = 145 children between 22 and 30 months old were recruited from the “Saving Brains Grenada” project and assessed on the INTER-NDA. A detailed description of this project and its methodology has been published elsewhere [36]. The children were recruited from communities across Grenada via a community based ECD outreach program called *The Roving Caregivers.* The sample size (n = 145) is consistent with that recommended by the Toolkit for Measuring Early Child Development in Low- and Middle-Income Countries [12]. The sample consisted of majority female (*n* = 53.8%), all were Afro-Caribbean, all but one spoke English as a native language, and the average child age was 26 months (SD = 2.21 months) (Table 3).

**Table 3:** Characteristics of internal consistency sample of child participants & their caregivers (n = 145)

Characteristic	N (%) or Mean (SD)
Child Sex	Female – 78 (53.8%) Male – 67 (46.2%)
Child Ethnicity	Afro-Caribbean – 145 (100%)
Child Native language	English – 144 (99.3%) Nigerian – 1 (0.7%)
Child Age (Months)	26 (2.21)
Maternal Education	Primary School – 30 (21.0%) Secondary School – 68 (47.5%) Tertiary – 45 (31.5%)
Maternal Age	18-24 – 32 (22.1%) 25-30 – 53 (36.5%) 31-38 – 47 (32.4%) Over 38 – 13 (9.0%)
Household Monthly Income (Eastern Caribbean Dollars – XCD)	Under $500 – 18 (12.8%) $500-$1000 – 59 (41.8%) $1000-$2000 – 31 (22.0%) $2000+ − 33 (23.4%)
Gestation (Weeks)	39.12 (2.26)
Complications During Birth	Yes – 12 (8.3%) No – 133 (91.7%)
Post-Birth Illness or Health Problems	Yes – 23 (15.9%) No – 122 (84.1%)

### Measures

#### The INTERGROWTH-21st project neurodevelopment assessment (INTER-NDA)

**T**he INTER-NDA is a comprehensive, rapid assessment of cognition, (fine and gross) motor skills, language and (positive and negative) behavior for children aged 22–30 months (Additional file 1) [14]. Its 37 items are administered using a combination of psychometric techniques (direct administration, concurrent observation and caregiver reports) in approximately 15 min. Children’s performance on the INTER-NDA is scored across a spectrum of abilities, rather than on a predefined checklist and, therefore, affords a wider description of a child’s faculties. It has been reported to have strong agreement with the Bayley Scales of Infant and Toddler Development III edition, (BSID-III) (interclass correlation coefficients 0·75–0·88, *p* < 0·001 for all domains with little to no bias on Bland Altman analysis); satisfactory internal consistency (Cronbach’s alpha 0.56–0.81) and good unidimensionality across subscales (Comparative Fit Index = 0.90; Tucker-Lewis Index = 0.94) and good levels of inter-rater (k = 0·70; 95% CI 0·47 to 0·88) and test–retest reliability (k = 0·79; 95% CI 0·48 to 0·96) [13, 24].

The INTER-NDA is designed for use across socioeconomic groups and populations. Its operation manual, standardization protocol and forms are freely available at www.intergrowth21.org.uk. The kit consists of common household items encountered across the world. The INTER-NDA was developed in 2014 by the International Fetal and Newborn Growth Consortium for the twenty-first Century (INTERGROWTH-21st) Project, a population-based, longitudinal study in five countries, including Brazil, India, Italy, Kenya, and the United Kingdom [35]. In all INTERGROWTH-21st Project study sites, the INTER-NDA was translated into the local languages of the sites using the WHO Mental Health Initiative translation guidelines, which included processes of cultural customization, translation and back translation [14]. The INTER-NDA’s norms are international standards (rather than population-specific references that are adapted for use in the Caribbean) for child development at 2 years of age, constructed from the INTERGROWTH-21st Project population using, the WHO’s prescriptive methodology. Scaled INTER-NDA domain scores are interpreted against the INTER-NDA’s standards to ascertain a child’s risk of no (>10th centile), mild-to-moderate (3rd to 10th centile) or severe (<3rd centile) neurodevelopmental delay for each of the domains [14].

#### Training of child development assessors

Seven non-specialist child development assessors were trained in the INTER-NDA in Grenada over a five-day period by the developer of the INTER-NDA (MF) who is a UK-based paediatrician, and who served as the expert assessor (expert) for the purpose of this study. During the training process, importance was paid to the conceptual basis of each item, the accurate administration of tasks to the child, as well as the accurate and objective reporting of the child’s performance on each item. All sessions were video recorded, and the expert observed the trainee-assessors carrying out three assessments each on 22-30-month-old children, randomly assigned. After these sessions, the expert provided each trainee-assessor with feedback on their administration and scoring of the INTER-NDA and on their interaction with the child and the caregiver.

#### INTER-NDA adaptation process to the Caribbean context

The process of the linguistic and cultural adaptation of the INTER-NDA was undertaken on day three of the training, after each trainee-assessor had assessed at least one child, and involved all trainee-assessors, the study PIs and the expert assessor. This process adhered to the previously published, recommended guidelines for the adaptation of an ECD instrument [11]. The process consisted of the following steps:

1. The trainee-assessors and PIs evaluated each of the 37 INTER-NDA items for linguistic and cultural relevance. Items considered to benefit from amendments to fit the local context were identified.
2. The trainee-assessors proposed alternatives to the phraseology of the items identified above. These options were discussed with the study PIs and expert assessor. Each option was scrutinized for contextual relevance by the trainee and expert assessors; any conflicts identified were discussed among all the assessors and alternative phraseology was proposed.
3. The eight adapted items were compared to the original INTER-NDA items by the expert assessor to confirm conceptual equivalence in accurately screening for NDIs. Conflicts identified were discussed with the trainee-assessors, alternative phraseology was considered and the process of assessment for contextual and conceptual equivalence repeated until a consensus was achieved.
4. The final list of adapted items were included into the measure and piloted on children aged 22-30 months.

#### Evaluation of the INTER-NDA’s administration and scoring by non-specialist child development assessors

Each non-specialist child developmental assessor was evaluated for their ability to (1) administer and (2) score the INTER-NDA in a standardized manner according the INTERGROWTH-21st Project protocols. To assess their ability to administer the INTER-NDA in a standardized manner, each non-specialist child development assessor was rated for protocol adherence on the INTERGROWTH-21st Project’s INTER-NDA protocol adherence checklist (Additional file 2; http://www.medscinet.net/Intergrowth/patientinfodocs/Standardisation%20Protocol.pdf) by the six other non-specialist child developmental assessors and the expert assessor. The agreement between each trainee-assessors’ INTER-NDA scores was compared with the expert’s INTER-NDA scores, across INTER-NDA domains, for the four video recordings of the INTER-NDA described in the reliability experiment above. Protocol adherence scores were summed across all the items to yield an overall INTER-NDA protocol adherence score for each trainee-assessor, from which protocol adherence percentages (range 0-100%) were calculated. The agreement between INTER-NDA domain scores, measured on the adapted INTER-NDA, was compared between trainee-assessors and the expert using kappa coefficients [6, 7].

#### Statistical analysis - assessment of psychometric properties of the adapted INTER-NDA

All data were analysed in SPSS Statistics v21.0.0.0©IBM Corp. Inter-rater and test-retest reliability between INTER-NDA scores across domains was determined for each trainee-assessor based on their scoring of four videos of the expert assessor administering the INTER-NDA to two-year old children. The second and fourth videos were identical, and trainee-assessors scored these videos separately, without access to the scores of video 1, allowing an assessment of test-retest reliability. Discussion between trainee-assessors and replaying of sections of the videos was not permitted. The video-based approach was selected over a conventional real-time approach to ensure that the child’s scores were not affected by: (i) anxiety about performance in the presence a large number of assessors; and (ii) inability of assessors to hear and see the child’s performance uniformly at all times during the assessment. This approach has been previously used in the INTERGROWTH-21st Project for reliability assessments [14, 24]. Each trainee-assessor administered the INTER-NDA on three children, randomly assigned, Test-retest and inter-rater reliability for the adapted INTER-NDA, across domains, was quantified using kappa coefficients [6, 7]. Kappas of 0.81 and above are considered representative of almost perfect agreement, kappas of 0.61 - 0.80 as substantial agreement, kappas of 0.41 - 0.60 as moderate agreement, kappas of 0.21 - 0.40 as fair agreement, and kappas of 0.20 and below as poor agreement [7]. Internal consistency was determined for each INTER-NDA domain by calculating Cronbach’s alphas on a separate group of *n* = 145 children between 22 and 30 months of age [4]. Cronbach’s alpha values were considered “good” if they were above a threshold of 0.7 [32].

#### Ethics

The Institutional Review Board at St. George’s University approved the study (IRB #14066). All research personnel involved in the study completed the National Institutes of Health Office of Extramural Research *Protecting Human Research Participants* online course. Parents/guardians provided informed written consent on behalf of the participating children. Participating child development assessors provided informed written consent.

## Results

### Adaptation of the INTER-NDA to the English-speaking Caribbean context

Following the evaluation of each of the 37 INTER-NDA items for linguistic and cultural relevance, eight items were identified to benefit from amendments to fit the local context. These were adapted to meet the study’s requirements of cultural and linguistic prevalence, and the resulting amendments were scrutinized for conceptual equivalence with the corresponding original items of the INTER-NDA by the expert. The adaptations, as well the justifications for these, are presented in Table 4.

**Table 4:** Adaptation of the INTER-NDA items to the Caribbean context

					Adaption meets criteria for:		
INTER-NDA item No.	Original INTER-NDA item	Adapted item to the Caribbean context	Rationale for amendment	Justification	Linguistic relevance	Cultural relevance	Conceptual equivalence
1.	Builds a tower of 5 **cubes** (Examiner says “Could you build a tower with the **cubes**?”)	Builds a tower of 5 **blocks** (Examiner says “Could you build a tower with the **blocks**?”)	Colloquialism - ‘**Cubes’** replaced by **‘blocks’**	‘**Cubes’** are referred to as **‘blocks’** in local vernacular	✓	✓	✓
4.	Hands the examiner one **cube** when asked to do so. (Examiner says “Please **give me** one **cube**” & keeps palm open for 5 s after child has handed over 1 cube)	Hands the examiner one **block** when asked to do so. (Examiner says “Please **hand me** one **block**” & keeps palm open for 5 s after child has handed over 1 block)”	Colloquialism - ‘Cubes’ replaced by ‘blocks’; **Give me**” is changed to “**Hand me**”	In local vernacular, many Grenadians ask others to “**hand me”** an object, as opposed to **“give me”** the object	✓	✓	✓
8.	Points correctly when asked “**Where is** the door/entrance to the room?”	Points correctly when asked “**Show me** the door/entrance to the room?”	Colloquialism - “**Where is**” is changed to “**Show me**”	Grenadians are often asked to “**show”** where an object is located, as opposed to being asked **“where”** the object is located			
12.	Able to make a cup of tea with the toy tea set when requested by examiner (Examiner says “Can you make a cup of tea?”)	Able to make a cup of tea with the toy kettle when requested by examiner (Examiner says “Can you make a cup of tea?”)	Cultural context - the **tea pot** is understood to be a “**kettle**” by the children.	Many Grenadians substitute a kettle for a teapot, as tea brewing is not a widely familiar practice on the island, especially among low-income families.	✓	✓	✓
14.	Feeds doll when requested to (Examiner says “Can you give the dolly some **tea**?”)	Feeds doll when requested to (Examiner says “Can you give the dolly some **milk**?”)	Cultural context - tea is understood to be **warm milk** or **baby formula**.	Grenadian children are not familiar with the concept of tea, as the term refers to any hot beverage—not solely tea as the rest of the world knows it. The age group of children tested in the study know “tea” to be warm milk.	✓	✓	✓
20.	Throws a ball very near (Examiner says “**Can you throw** the ball?”)	Throws a ball very near (Examiner says “**Can you pelt** the ball?” or “**Pelt the ball for me**” or “**Send the ball for me**”)	Colloquialism - “**throw**” is changed to “**pelt**” or “**send**”	When asked using the former phrase, Grenadian children would gently drop the ball in front of them. Children would respond to “pelt the ball” by throwing the ball overhand.	✓	✓	✓
26.	Use of a pronoun e.g. [**mine**] me, my, she, he, it, I (Examiner says “**Who does this belong to? “Whose is this**?”)	Use of a pronoun e.g. [**mines**] me, my, she, he, it, I (Examiner says “**Who dat/dis for**?”)	Colloquialism - “**mine**” is changed to “**mines**” and “**Whose is this**?” changed to “**Who dat/dis for**?”	Many Grenadians utilize **“dat”** and/or **“dis”** in accordance with local vernacular. Additionally, phonetic differences between singular and plural words is not always observed in local Grenadian vernacular.	✓	✓	✓
30.	Combines word and gesture when asked (examples: high-five)	Combines word and gesture when asked (example: bounce)	Change to examples of word-gesture combinations used in the item; (most common gesture is “**a bounce**” rather than a “**high-five**” or “**flying kiss**”)	Grenadian children are more familiar with a **“bounce”** than a **“high five”** – the former is a gesture where both individuals make a fist and tap their knuckles together.	✓	✓	✓

### Psychometric properties of the adapted INTER-NDA

The inter-rater and test-retest reliability across all domains of the INTER-NDA, as determined by the assessments of *n* = 21 children by the seven non-specialist child developmental assessors, is presented in Table 5. Inter-rater reliability and test-retest reliability among the seven assessors ranged between κ = 0.99 - 1.00, 95% CI [0.98, 0.99] and κ = 0.76 - 1.00 (95% CI [0.33, 1.00]) respectively, across all domains of the INTER-NDA, representing fair to near-perfect agreement [7]. The Cronbach’s alpha scores for the sample of *n* = 145 children are presented in Table 6. These were near-perfect for the cognitive (α = 0.84), language (α = 0.84) and positive behavior (α = 0.90) domains, substantial for the fine motor (α = 0.62) domain, and fair for the negative behavior (α = 0.31) domain. The Cronbach alpha score is not reported for the gross motor domain because one of the 3 items that make up this domain showed ceiling effects, greatly reducing confidence in the accuracy of the score. The corresponding internal consistencies of the original INTER-NDA, previously published, are also presented in Table 6 for comparison [24].

**Table 5:** Reliability assessments

INTER-NDA Subscale	Inter-rater Reliability	Test-retest Reliability	Agreement between non-specialist assessors and expert			
	κ	95% CI	κ	95% CI	κ	95% CI
Cognition	1.00	–	0.97	[0.76, 1.00]	0.96	[0.71, 1.00]
Language	0.99	[0.98, 0.99]	0.81	[0.61, 1.00]	1.00	–
Gross Motor	1.00	–	1.00	–	0.94	[0.58, 1.00]
Fine Motor	1.00	–	0.94	[0.56, 1.00]	1.00	–
Positive Behavior	0.99	[0.98, 0.99]	1.00	–	0.92	[0.59, 1.00]
Negative Behavior	1.00	–	0.81	[0.33, 1.00]	κ	95% CI
Total INTER-NDA	0.99	[0.98, 0.99]	0.86	[0.74, 1.00]	0.90	[0.64, 1.00]

κ = kappa coefficient, CI = confidence interval, CIs not reported for domains that show perfect agreement (κ = 1.00)

**Table 6:** Internal consistencies of the adapted INTER-NDA

INTER-NDA domain	Caribbean Adaptation	Original INTER-NDA^a^
Cognition	0.84	0.81
Language	0.84	0.83-0.90
Gross Motor	Not reported^b^	Not reported
Fine Motor	0.62	Not reported
Positive Behavior	0.90	0.85
Negative Behavior	0.31	0.56

^a^Murray, E., Fernandes, M., Newton, C. R. J., Abubakar, A., Kennedy, S. H., Villar, J., & Stein, A. (2018) Evaluation of the INTERGROWTH-21st Neurodevelopment Assessment (INTER-NDA) in 2 year-old children. *PLoS ONE, 13*(2): e0193406. 10.1371/journal.pone.0193406

^b^Internal consistency is not reported for the gross motor domain because 1 of 3 items in that domain showed a ceiling effect

### Evaluation of the INTER-NDA’s administration and scoring by non-specialist child development assessors

The protocol adherence scores of non-specialist child development assessors in the context of the administration of the adapted INTER-NDA ranged between 70.5 and 100% (*n* = 7, *M* = 89.9%, *SD* = 10.1%) (Table 7).

**Table 7:** Protocol Adherence Scores for non-specialist child developmental assessors for the adapted INTER-NDA

Assessor Number	% Adherence
1	94.1
2	82.4
3	94.4
4	94.1
5	100
6	94.1
7	70.5

Agreement between the expert and the non-specialist child development assessors is presented in Table 5 and ranged between κ = 0.89 and k = 1.00 (95% CI 0.58, 1.00) representing almost perfect agreement [7].

## Discussion

This study presents, to our knowledge, the first adaptation of the INTER-NDA to the linguistic and cultural context of the Caribbean, making the INTER-NDA the first ECD measure specifically adapted for use in the English-speaking Caribbean. The adapted INTER-NDA is very similar to the original INTER-NDA, with linguistic and cultural adaptations in 8 of the 37 items while simultaneously maintaining conceptual equivalence between the original and adapted versions. The inter-rater and test-retest reliability of the adapted INTER-NDA, and its internal consistency, were satisfactory across domains and comparable to the original INTER-NDA. Importantly, especially in the context of LMIC settings, we also showed that the adapted INTER-NDA can be administered and scored by non-specialist child development assessors at high levels of protocol adherence and agreement with an expert specialist assessor.

The results of this study provide evidence to support the use of the adapted INTER-NDA to measure ECD prevalence in the English-speaking Caribbean and for non-specialist child development assessors, trained and standardized in the INTER-NDA, to use the tool to conduct ECD evaluations. Previous studies have shown that the INTER-NDA can be administered in school settings in Mexico, and in research settings in Brazil, India, Italy, Kenya and the UK, by non-specialist assessors at high levels of reliability and protocol adherence [14, 31]. These studies have focused on the translation, back translation and cultural adaptation of the INTER-NDA, developed initially in British English, into non-English languages. This study extends this work by culturally and linguistically adapting the INTER-NDA for use in an English-speaking, yet culturally and geographically diverse, LMIC population, i.e. the English-speaking Caribbean. Moreover, we have presented a methodological template for this process, which we hope can be applied to the adaptation and subsequent pilot testing of other ECD tools for use in various Caribbean settings.

### Strengths and limitations of the study

This study is important in a number of ways. First, consideration was given to different lexical items to express similar concepts in British and Creole English; for example, “throw” and “pelt”; “cubes” and “blocks”; and “teapot” and “kettle. This is particularly necessary in the Grenadian context where children are more familiar with Creole English, rather than British English, during early life. Second, care was taken to ensure that the components of the INTER-NDA’s kit were familiar to Caribbean children, and commonly encountered in Caribbean households. The use of items unfamiliar to the average child’s repertoire of household and play-related exposures, such as a puffed rice grain or a maize bean [16, 19]; or items which the child may be forbidden from playing with, such as a matchbox [16], are commonly overlooked factors that can skew ECD assessment results. Third, we assessed whether non-specialist child development assessors, in the Caribbean context, can measure ECD outcomes on the adapted INTER-NDA. In LMIC settings where reliance for ECD assessments on specialist professionals for their administration, scoring and interpretation is one of the key rate-limiting steps to the scalability of ECD surveillance and for the identification of children at risk who may benefit from interventions. Although non-specialists have been previously shown to administer and score the INTER-NDA reliably, this study is, to our knowledge, the first effort to assess this in a Caribbean setting and provides evidence to support ECD evaluations in Grenada by non-specialist child development assessors.

There are a number of limitations to consider. First, the sample size used to determine inter-rater reliability and test-retest reliability was small – 7 trainee-assessors performed 3 randomly-assigned child assessments each, for a total of 21 assessments. Thus, test-retest reliability and inter-rater reliability statistics should be interpreted with caution. Second, this study was carried out in Grenada, and while socio-cultural similarities exist among Caribbean nations, the adapted INTER-NDA should be piloted and assessed in other Caribbean settings to establish firmer reliability. Third, this adaptation of the INTER-NDA is limited to the English-speaking Caribbean, despite substantial proportions of the region’s population being native in Spanish and French. Fourth, our results are restricted to the INTER-NDA’s age range of assessment (22 to 30 months) and, as such, ECD tools for the assessment of a wider age range of children should be adapted and standardized in order to significantly impact ECD surveillance in the Caribbean across the early childhood. Fifth, we were not able to assess internal consistency of the gross motor domain within the INTER-NDA as a result of measurement challenges (i.e., ceiling effects) with one of the 3 items that make up this domain score.

### Context of the study

An essential condition for achieving the United Nations Sustainable Development Goal (UN SDG) 4.2 (‘ensure that all girls and boys have access to quality early child development, care and preprimary education so that they are ready for primary education’) [33] is the measurement of ECD outcomes at scale, in order to identify children at risk and to make cross-population comparisons [14]. While a multitude of tools to measure ECD risk exist, none are culturally adapted and standardized for use in the Caribbean setting, especially for administration by non-specialists. By linguistically and culturally adapting the INTER-NDA to the context of the English-speaking Caribbean, without compromising its conceptual integrity, we have, for the first time, produced a practically applicable, culturally relevant ECD measure specifically adapted to this setting. The INTER-NDA is a unique clinical tool for use across all healthcare systems to measure neurodevelopmental milestones and associated behaviors in 2-year-olds uniformly and at scale, and to identify children at risk of NDIs who would benefit from specialist referral and further investigation [14]. It is our hope that the adapted INTER-NDA will complement the INTERGROWTH-21st Project’s international INTER-NDA standards [14] for the measurement of ECD outcomes in the Caribbean while simultaneously providing a methodological template for the adaptation of child developmental measures to cultural and linguistic contexts.

### Conclusion

This study represents, to our knowledge, the first adaptation of an ECD instrument to the English-speaking Caribbean. This is important for standardized, robust ECD measurement in the region, at scale, and supports the inclusion of Caribbean infants in international efforts at ECD surveillance. Furthermore, this study highlights the importance of the linguistic and cultural adaptation of ECD measures, even between settings which appear to share the same primary language but in which social, cultural, geographic and economic contexts vary.

## Supplementary Information

**Additional file 1.** The INTERGROWTH-21^st^ Neurodevelopment Assessment (INTER-NDA).

**Additional file 2.** The INTERGROWTH-21^st^ Neurodevelopment Assessment (INTER-NDA) Protocol Adherence Checklist.

## Data Availability

The data that support the findings of this study are available from the corresponding author (SH) upon reasonable request.

## Abbreviations

INTER-NDA: The INTERGROWTH-21st Neurodevelopment Assessment
ECD: early child development
LMICs: low and middle-income countries
NDI: neurodevelopmental impairment
BSID-III: Bayley Scales of Infant and Toddler Development III edition
*SD*: standard deviation
UN SDG: United Nations Sustainable Development Goal
