# Supplementary material for: Adaptation of the INTERGROWTH-21st neurodevelopment assessment (INTER-NDA) to the context of the English-speaking Caribbean
Source: BMC Pediatr. 2022 Jan 4;22:21. doi: 10.1186/s12887-021-03039-7 (PMC8728897; doi:10.1186/s12887-021-03039-7)

**Appendix A**

*The INTERGROWTH-21st Neurodevelopment Assessment (INTER-NDA)*

*(*Reproduced with permission from Fernandes et al. (2020). The INTERGROWTH-21^st^ Project Neurodevelopment Assessment (INTER-NDA) data recording form (S4). *BMJ Open, 10*(6), e035258. doi:10.1136/bmjopen-2019-035258)


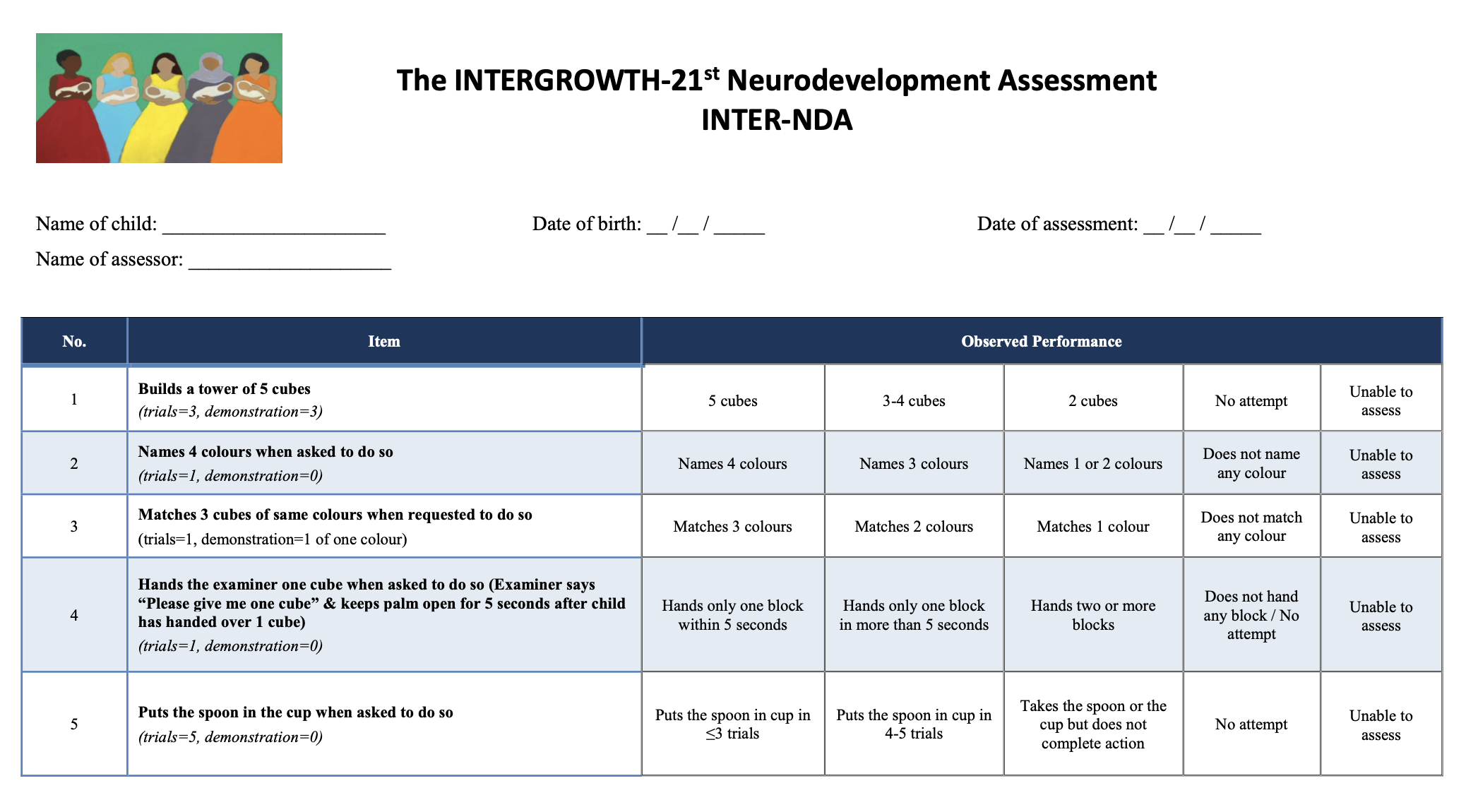


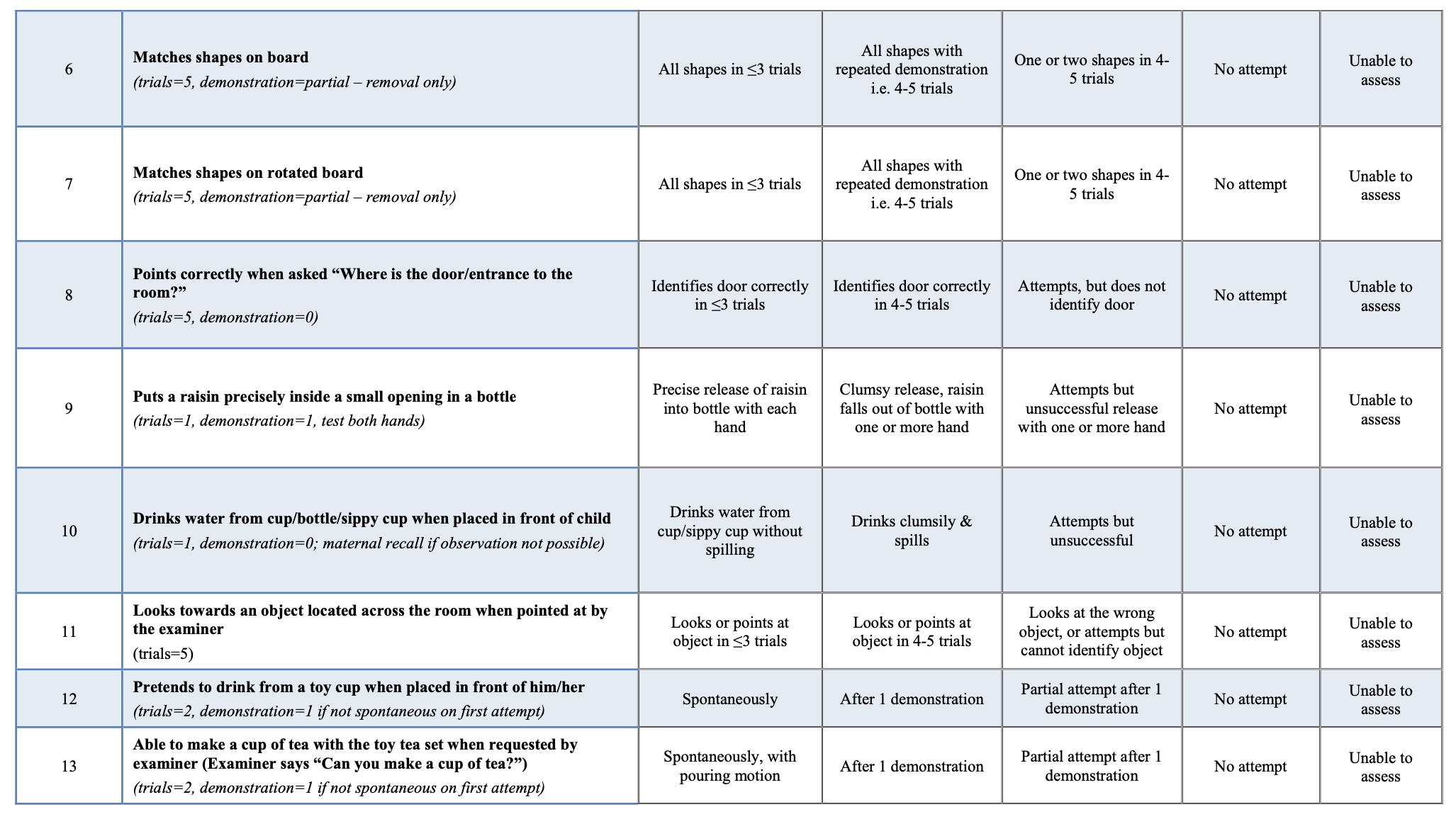


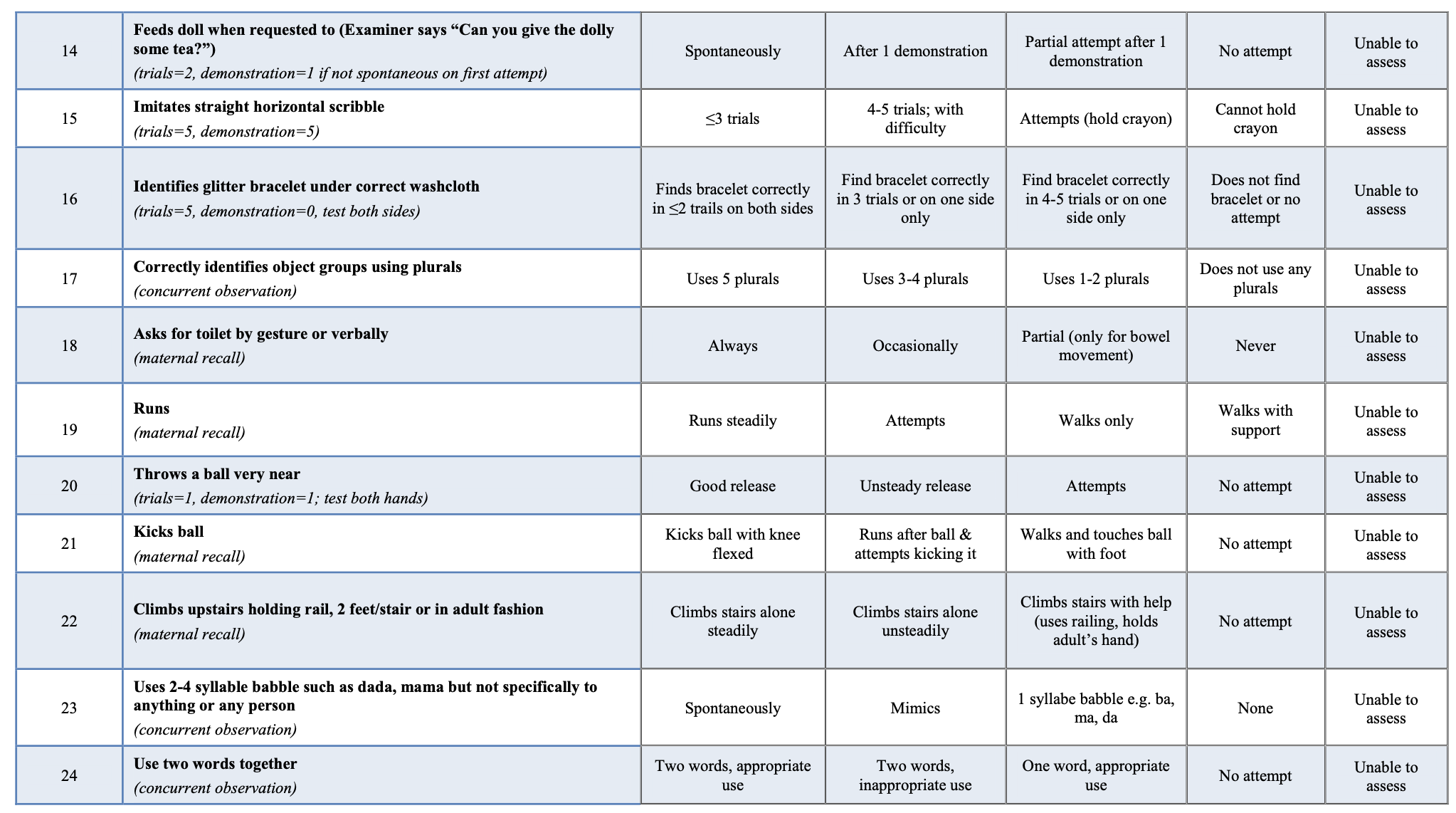


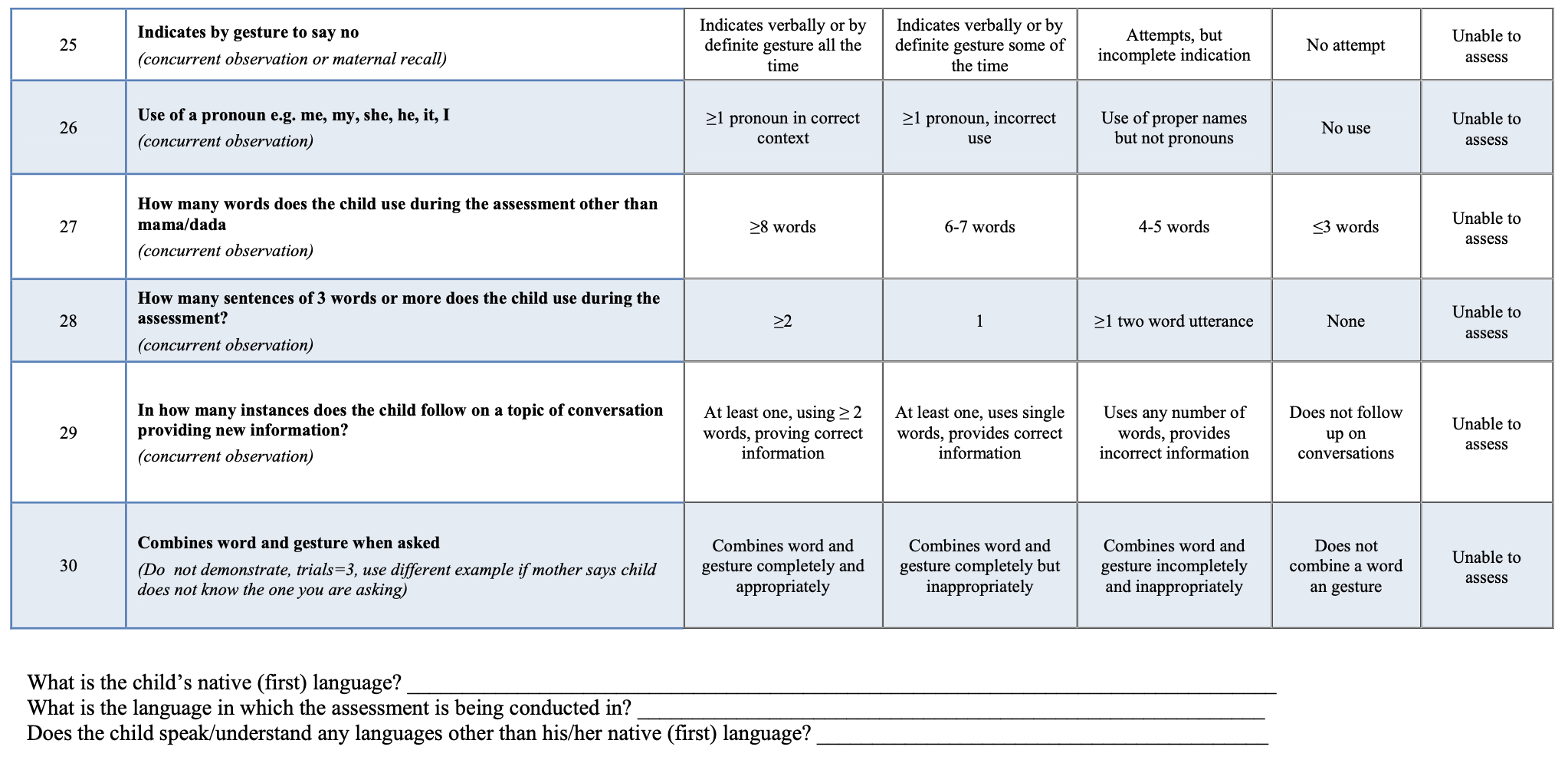


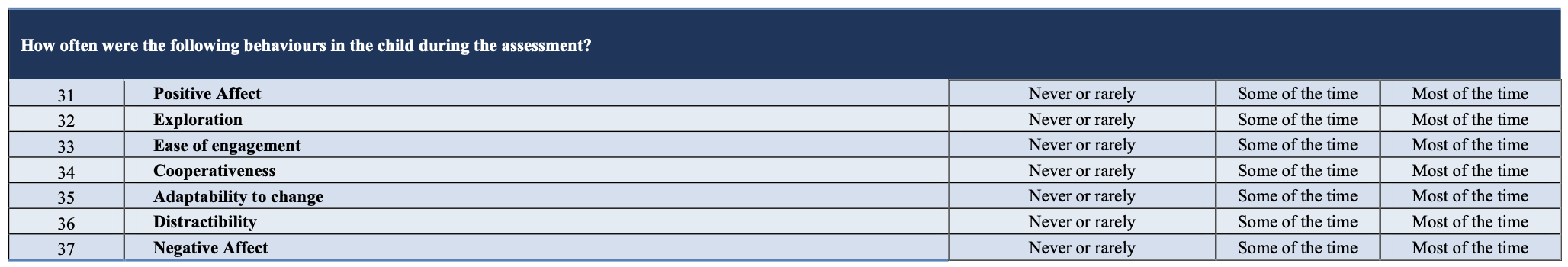

Supplement: Supplementary file 1 — Additional file 1. The INTERGROWTH-21st Neurodevelopment Assessment (INTER-NDA). [file 12887_2021_3039_MOESM1_ESM.docx]
