# Supplementary material for: Adaptation of the INTERGROWTH-21st neurodevelopment assessment (INTER-NDA) to the context of the English-speaking Caribbean
Source: BMC Pediatr. 2022 Jan 4;22:21. doi: 10.1186/s12887-021-03039-7 (PMC8728897; doi:10.1186/s12887-021-03039-7)

**Appendix B**

The INTERGROWTH-21st Neurodevelopment Assessment (INTER-NDA) Protocol Adherence Checklist

(Reproduced with permission from Savini S et al. (2014). The INTERGROWTH-21^st^ Project Neurodevelopment Package Standardization Protocol v.6.; http://www.medscinet.net/Intergrowth/patientinfodocs/Standardisation%20Protocol.pdf)


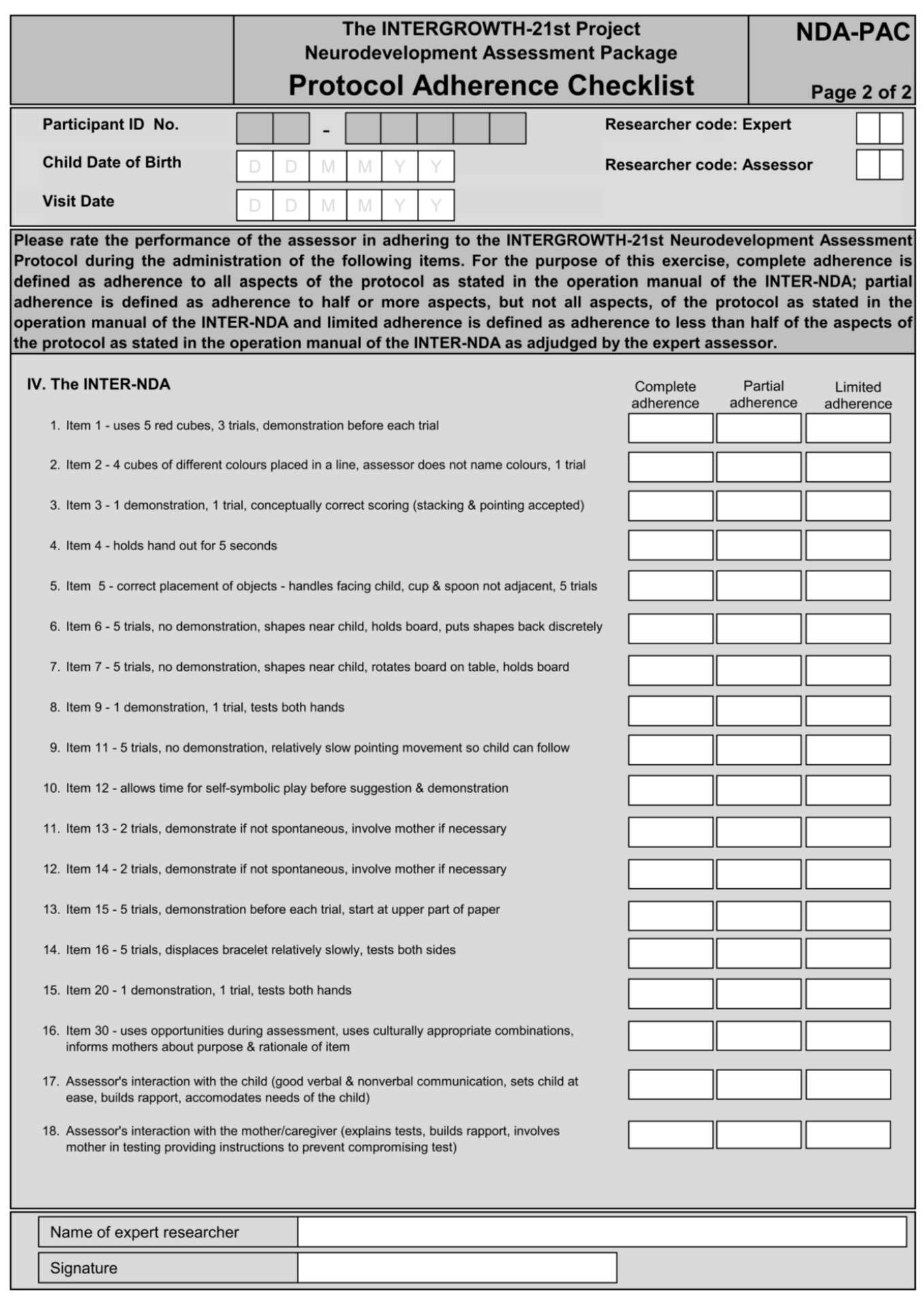

Supplement: Supplementary file 2 — Additional file 2. The INTERGROWTH-21st Neurodevelopment Assessment (INTER-NDA) Protocol Adherence Checklist. [file 12887_2021_3039_MOESM2_ESM.docx]
